# Supplementary material for: The Morphological and Anatomical Traits of the Leaf in Representative Vinca Species Observed on Indoor- and Outdoor-Grown Plants
Source: Plants (Basel). 2021 Mar 24;10(4):622. doi: 10.3390/plants10040622 (PMC8064346; doi:10.3390/plants10040622)
Supplement: Supplementary file 1 [file plants-10-00622-s001.pdf]

Supporting Information

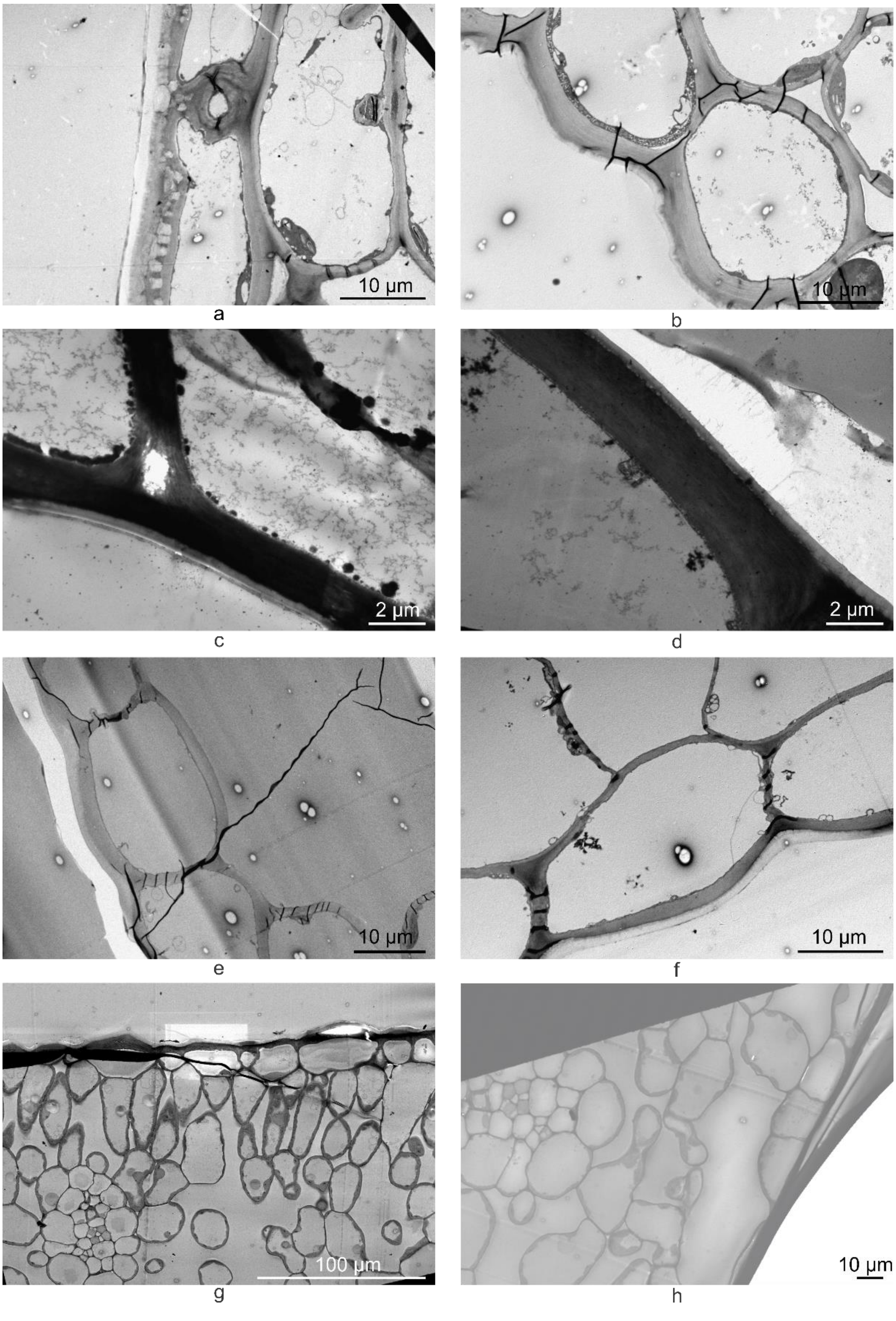

Figure S1. TEM micrographs of the zoomed-out cell walls of *V. minor* (**a–b**), *V. major* (**c–d**), *V. major* var. *variegata* (**e–f**), and *V. herbacea* (**g–h**). In the first column, the upper epidermis is presented and the second column, the lower epidermis.
